# Supplementary material for: Circulating miRNAs Expression in Myalgic Encephalomyelitis/Chronic Fatigue Syndrome
Source: Int J Mol Sci. 2023 Jun 24;24(13):10582. doi: 10.3390/ijms241310582 (PMC10341915; doi:10.3390/ijms241310582)
Supplement: Supplementary file 1 [file ijms-24-10582-s001.zip › ijms-2452271-supplementary.pdf]

Supplementary

Table S1. Correlation between inflammatory cytokines, HHV-6A/B presence and miRNAs expression.

	IFN-y		IL-17A		IL-2		IL-21		IL-23		IL-6		TNF-a	
	r[1]	*p*-value	r[1]	*p*-value	r[1]	*p*-value	r[1]	*p*-value	r[1]	*p*-value	r[1]	*p*-value	r[1]	*p*-value
**miR-124-3p**	-0,067	0,613	-0,092	0,487	-0,022	0,866	0,000	0,997	0,016	0,905	0,092	0,500	0,061	0,646
**miR-140-5p**	-0,066	0,622	0,069	0,606	0,007	0,957	0,170	0,197	0,094	0,479	0,033	0,810	0,157	0,236
**miR-551b-3p**	-0,161	0,223	-0,188	0,154	-0,249	0,057	-0,098	0,462	-0,124	0,351	-0,134	0,327	-0,027	0,841
**miR-448**	-0,271	0,038	-0,239	0,068	-0,259	0,048	-0,438	0,001	-0,366	0,004	-0,379	0,004	-0,232	0,077
**miR-142-5p**	-0,245	0,061	-0,133	0,315	-0,114	0,391	-0,152	0,250	-0,178	0,176	0,006	0,962	-0,066	0,617
**miR-127-3p**	-0,031	0,815	0,080	0,545	-0,014	0,916	0,083	0,533	0,097	0,465	0,061	0,656	0,170	0,197
**miR-143-3p**	-0,303	0,020	-0,169	0,201	-0,199	0,131	-0,155	0,242	-0,257	0,050	-0,179	0,188	0,008	0,950
**miR-150-5p**	-0,214	0,104	-0,145	0,272	-0,183	0,165	-0,178	0,177	-0,215	0,102	-0,325	0,015	-0,124	0,350
**HHV-6 copies/10^6 cells**	-0,121	0,362	-0,122	0,358	-0,083	0,534	-0,144	0,275	-0,060	0,651	-0,040	0,768	-0,181	0,170

[1] Correlation coefficients (r) obtained from Spearman Correlation analysis.
